# Switchable Retargeting of Lentiviral Vectors Through a VSV-G-Binding Adapter Molecule

**DOI:** 10.3390/v17121563

**Published:** 2025-11-29

**Authors:** Vladislav A. Zhuchkov, Marat P. Valikhov, Yulia E. Kravchenko, Elena I. Frolova, Stepan P. Chumakov

**Affiliations:** 1 Shemyakin-Ovchinnikov Institute of Bioorganic Chemistry, Russian Academy of Sciences, 117997 Moscow, Russia; vladik55318@gmail.com (V.A.Z.);; 2 Biomarker Research Laboratory, Institute of Fundamental Medicine and Biology, Kazan Federal University, 420008 Kazan, Russia; 3 Engelhardt Institute of Molecular Biology, Russian Academy of Sciences, 119991 Moscow, Russia; marat.valikhov@gmail.com

**Keywords:** lentivector, retargeting, nanobody, VHH, VSV-G, ERBB2

## Abstract

Selective gene delivery to defined cell populations remains one of the key challenges in lentiviral vector-based gene therapy. The vesicular stomatitis virus glycoprotein (VSV-G) confers high infectivity but lacks cell-type specificity because of the ubiquitous expression of its receptor, LDLR. To enable modular, receptor-specific targeting while retaining the production efficiency of VSV-G-pseudotyped vectors, we designed a bispecific adapter, 929-B6, comprising a VSV-G-binding nanobody and an ERBB2-binding DARPin 9.29. Anti-VSV-G nanobodies were isolated from an alpaca immune library and screened in cell-based pseudoreceptor assays to identify the optimal binder (VSVG-B6). The resulting adapter was evaluated with receptor-ablated (VSV-G_mut_) and wild-type VSV-G-pseudotyped LVs across ERBB2-positive and -negative cell lines and in a mouse xenograft model. 929-B6 enabled efficient, receptor-specific transduction of ERBB2-expressing cells without increasing infection of ERBB2-negative controls. Pre-incubation of VSV-G_mut_-pseudotyped LVs with 1–2 µg/mL 929-B6 increased transduction up to eight-fold in ERBB2^+^ cells, with similar but smaller effects for VSV-G and VSV-G_mut_ + 929R pseudotypes. Across breast cancer lines, transduction enhancement correlated with ERBB2 surface density, and co-culture experiments confirmed selective entry into ERBB2^+^ populations. In vivo imaging of ERBB2^+^ tumors revealed a visible tumor-localized luminescent signal following administration of 929-B6-treated vectors. The 929-B6 adapter provides a rapid, scalable means to retarget standard LV stocks toward chosen receptors without re-engineering the envelope or co-packaging pseudoreceptor plasmids. Its modularity suggests a generalizable platform for both gene therapy and oncolytic applications requiring flexible, receptor-defined tropism.

## 1. Introduction

Targeted delivery of transgenes into specific cell populations using lentiviral vectors represents a powerful and promising approach for gene therapy of hereditary and oncological diseases. The most commonly used glycoprotein for pseudotyping lentiviral vectors, the vesicular stomatitis virus glycoprotein G (VSV-G), does not allow selective targeting of distinct cell populations due to the nearly ubiquitous expression of its cellular receptor, the low-density lipoprotein receptor (LDLR) [1].

Efforts to improve the specificity of lentiviral vectors have led to the development of several receptor retargeting strategies aimed at redirecting viral entry toward defined cell populations [2]. One of the earliest approaches involved genetic fusion of targeting ligands or antibody fragments to the LV envelope protein, most often to modified forms of the Sindbis or MLV envelopes, enabling antibody-mediated or ligand-specific transduction of selected cells [3]. ScFv-VSV-G fusion chimeras have been used to graft antigen specificity onto virus particles, though often at the cost of reduced fusogenicity [4]. Another direction has been pseudotyping LVs with heterologous or chimeric envelopes from viruses that possess natural tropism for particular tissues [5], such as measles [6] or Nipah [7] virus glycoproteins, to exploit their receptor specificity while maintaining LV backbone compatibility; however, while pseudotyping with alternative viral glycoproteins can achieve a narrower cell-type specificity, the tropism is limited by that of the original virus. The generation of receptor-blinded versions of paramyxoviral glycoproteins, such as those of the measles virus [8] and Nipah [9], Tupaia [10], or CDV [11], enabled the engineering of modified H and G glycoproteins whose tropism is determined by an introduced receptor-binding domain, such as a DARPin [12], scFv [13], nanobody [14], or a natural ligand [15] of a cellular receptor. Lentiviral vectors pseudotyped with such engineered glycoproteins selectively target cells expressing the cognate receptor recognized by the introduced binding domain. Although this strategy enables highly specific transduction, these lentiviral particles are considerably more difficult to produce: infectious titers of lentivectors pseudotyped with paramyxoviral glycoproteins are typically at least one order of magnitude lower than those of VSV-G-pseudotyped vectors [16], and retargeted variants generally exhibit titers an additional order of magnitude lower [17].

Recently, the crystal structure of the VSV-G-LDLR complex was resolved, revealing that VSV-G binds to two adjacent CR2 and CR3 domains of LDLR; two basic residues (K47 and R354) are essential for this interaction, and their substitution (K47Q, R354A) yields an LDLR-blind variant, termed VSV-G_mut_ [18]. Importantly, VSV-G not only confers receptor binding, but also drives efficient internalization and fusion: after receptor engagement, virions are internalized by clathrin-mediated endocytosis, and as endosomes mature and acidify, the low pH triggers a conformational change in the G protein that drives membrane fusion, releasing viral contents into the cytosol [19]. This two-step route of entry is preserved when VSV-G is used to pseudotype lentiviral particles, contributing to the high infectivity and stability of VSV-G-pseudotyped LVs [20]. The efficiency and robustness of this mechanism help explain why alternative glycoproteins often struggle to reach comparable titers when retargeted: many engineered or chimeric envelopes falter in one or more steps (binding strength, internalization, fusion, or incorporation) and fail to match VSV-G’s performance in the stringent biological bottlenecks of vector production and cell entry [21].

On this structural and mechanistic foundation, a retargeting strategy was proposed: co-expression in producer cells of a membrane-anchored “pseudoreceptor” bearing a defined antigen-recognition domain that binds the desired cellular receptor, together with VSV-G_mut_ [22]. Because VSV-G_mut_ no longer binds LDLR, binding specificity can be rerouted via the pseudoreceptor. This concept had been earlier demonstrated in the context of the measles virus H glycoprotein, where receptor binding and membrane fusion were functionally decoupled between two proteins [23]. When the pseudoreceptor is expressed in packaging cells, it decorates budding virions and redirects vector tropism; retargeting via co-expression of VSV-G_mut_ and pseudoreceptor can yield lentivectors with infectious titers only modestly lower than those of conventional VSV-G-pseudotyped vectors.

Because the genetic sequence encoding a targeting pseudoreceptor can be incorporated directly into the lentiviral genome, the resulting particles acquire a tropism genetically linked to their own genome [24]. This makes such pseudotyping systems especially useful for high-throughput selection of targeting variants from libraries of natural ligands, antibody fragments [22], or T-cell receptor domains [25].

However, when lentiviral targeting is applied not for selection but for direct gene delivery, the requirement for a separate construct encoding the pseudoreceptor becomes a limitation. The inclusion of an additional helper plasmid in the packaging mix reduces overall production efficiency [26], and because the pseudoreceptor is uniformly distributed over the cell surface rather than concentrated at budding sites, its incorporation into virions may be insufficient for achieving optimal infectivity [20].

Beyond genetically encoded strategies, chemical [27] or peptide-based retargeting systems have also been tested. Biotin–streptavidin adapters were developed, allowing to bridge the LV surface and target receptors [28]. Plug-and-play covalent adapter systems, such as engineered protein–peptide pairs that form disulfide bonds, have been used to functionalize virions post-assembly without altering envelope genes directly [29]. Bispecific antibody adaptors have been deployed to link de-targeted viral envelopes to target cell receptors, particularly for LVs pseudotyped with Sindbis envelope [30]. Similarly, measles glycoprotein-based adapter-based LV targeting has been reported as a strategy to enhance the safety and specificity of cellular therapies [31].

Switchable targeting systems could provide an edge for translational gene therapy and oncolytic vector applications. Tumor-targeted gene delivery mediated by lentiviral vectors could benefit from adaptively swapping targeting domains to match heterogeneous antigen expression in cancer. In adoptive cell therapies, such as CAR-T and genetically-modified HSCs, a modular LV targeting system might help with cell-type-restricted gene expression or in vivo retargeting of transduced cells. The flexibility to interchange adapters may accelerate preclinical screening, reduce the need to re-engineer entire viral envelopes for each new target, and simplify regulatory adaptation for patient-personalized constructs.

We hypothesized that using a modular adapter molecule, capable of simultaneously binding VSV-G_mut_ and the target cellular receptor, would combine the strengths of these prior methods, such as flexibility and modular targeting, without their downsides. In contrast to scFv-VSV-G fusions or covalent post-assembly adapters, our approach retains standard LV packaging workflows, avoids the need for engineered envelope fusions for each target, and allows tropism switching simply by substituting the adapter. To evaluate this concept in practice, we chose ERBB2 as the model receptor and employed DARPin 9.29 [32] as a high-affinity targeting domain.

## 2. Materials and Methods

### 2.1. Plasmids and Cloning

The pLCMV-tagRFP, pLCMV-tagGFP2, pLCMV-fLuc, and pLCMV-nanoLuc [33] lentiviral vector expression plasmids were constructed previously. The psPAX2 and pMD2.G were a gift from Didier Trono (Addgene plasmid # 12260 and 12259). pHAGE-ERBB2 was a gift from Gordon Mills & Kenneth Scott (Addgene plasmid # 116734). pMD2.G_mut_ was constructed by introducing point mutations in VSV-G sequence by performing overlap-extension PCR on pMD2.G with primers VSVG EcoRi dir (GCACGTGAGATCTGAATTCAACAGAGATCG), VSVG 4147 dir (GGCACAGCCTTACAAGTCAAAATGCCCCAGAGTCACAAGG), VSVG 4147 rev (CCTTGTGACTCTGGGGCATTTTGACTTGTAAGGCTGTGCC), VSVG 354 dir (CAGTGGAACTACCACAGAAGCCGAACTGTGGGATGACTGG), VSVG 354 rev (CCAGTCATCCCACAGTTCGGCTTCTGTGGTAGTTCCACTG), and VSVG EcoRI rev (GCACTGGTGGGGTGAATTCC) and re-cloning PCR product digested by EcoRI into pMD2.G. pCG-929R was constructed by cloning DARPin 9.29 coding sequence into pCG-VHHR, which was constructed previously [14]. Lentiviral vector constructs for nanobody pseudoreceptor expression were constructed by subcloning Kozak and CD8 leader sequence (GCCACCATGGCCTTACCAGTGACCGCCTTGCTCCTGCCGCTGGCCTTGCTGCTCCACGCCGCCAGGCCG) followed by XbaI and BamHI recognition sites, a 12-amino acid spacer sequence G-GAGAGCAAGTACGGACCACCATGTCCACCATGCCCG, and the CD28 transmembrane domain sequence followed by the NheI recognition site (TTTTGGGTGCTGGTGGTGGTTGGTGGAGTCCTGGCTTGCTATAGCTTGCTAGTAACAGTGGCCTTTATTATTTTCTGGGTGGCTAGCAGGAGTAAGAGG), which was extended on its 5′-terminus by means of PCR amplification with primers CD8s BglII dir (AGAGAGAGATCTGCCACCATGGCCTTACCAGTGACCG) and CD28TM NheI rev (CCTCTTACTCCTGCTAGCCACCC), and digested with BglII and NheI into pLCMV-PL4-puro. pET22-929-VSVGB6 was constructed by first subcloning the VSVGB6 nanobody sequence via XbaI and BamHI restriction sites into the pET22-BAD plasmid vector, and then inserting the DARPin 9.29 sequence from pCG-929R, digested with XbaI and AvrII, via the XbaI restriction site.

### 2.2. Cells and Culturing

BHK-21 (CCL-10), HCC1954 (CRL-2338), SK-OV-3 (HTB-77), BT-474 (HTB-20), and IMR-32 (CCL-127) cell lines were purchased from ATCC (Manassas, VA, USA). HEK-293LTV cell line was purchased from Cell Biolabs (San Diego, CA, USA). EMT-ERBB2 cells (mouse mammary cancer cell line EMT6/P, stably expressing human HER2 gene [34]) were from the Shemyakin-Ovchinnikov Institute collection. All cell lines were cultured in DMEM/F12 media (Paneco, Moscow, Russia). The culture medium contained 10% fetal bovine serum (FBS; Gibco, Waltham, MA, USA), penicillin–streptomycin, L-glutamine, and non-essential amino acids (Gibco, Grand Island, NY, USA).

### 2.3. Transfection and Lentivirus Packing

All of the lentivirus-packaging transfections were performed with polyethyleneimine 25k (Polysciences, Warrington, PA, USA) according to the recommendations provided in [34]. The ratios of the plasmids for the lentivirus vector packaging were 5:3:2 (lentivector:psPAX2:pMD2.G or pMD2.G_mut_), or 5:3:2:3 (lentivector:psPAX2:pMD2.G_mut_:pCG-929R). For the syncytia assay, the ratio was 1:1 (pLCMV-tagGFP2:pMD2.G or pMD2.G_mut_). For lentivirus vector production, the day after transfection, the medium was changed to DMEM/F12 supplemented with PeproGrow serum replacement solution (Peprotech, Rocky Hill, NJ, USA), and the virus-containing medium was later collected after 48 h of incubation, filtered through a 0.45 μm syringe filter, and used for the transduction of recipient cells. To estimate viral titer, p24 concentrations in virus-containing media were measured with the Lenti-X p24 ELISA kit (Takara Bio, Mountain View, CA, USA). The equivalent of 2 × 10^3^ pg of p24 (~20 μL of viral media) per 1 × 10^5^ cells was used for transductions. Transductions were carried out in 6-, 12-, or 24-well plates for 120 min at 37 °C and 5% CO_2_; no transduction enhancers were utilized. For large-scale LV preparation, transfections were carried out in 5-layer cell culture flasks (Wuxi NEST Biotechnology, Wuxi, China) as previously described [33].

### 2.4. Generation of the HEK-ERBB2 Cell Line

A stable HEK-ERBB2 cell line was generated by transducing HEK-293LTV cells with a lentiviral vector encoding human ERBB2 (pHAGE-ERBB2). Transductions were performed as described above, using VSV-G-pseudotyped lentiviral particles. At 48 h post-transduction, cells were selected with puromycin (1 µg/mL) for 14 days to establish a population with stable ERBB2 expression. Surface expression of ERBB2 was verified by flow cytometry using CFL555-labeled anti-ERBB2 antibodies.

### 2.5. Antibodies and Flow Cytometry

Goat anti-human ErbB2/Her2 antibody AF1129 (R&D Systems, Minneapolis, MN, USA) and mouse anti-goat IgG-CFL 555 (Santa Cruz Biotechnology, Dallas, TX, USA) were used for ERBB2 staining on breast cancer cell lines. For ERBB2 detection in cell mixtures, trastuzumab (Biocad, Moscow, Russia) conjugated with AlexaFluor 488 (Thermo, Waltham, MA, USA) was utilized. All stainings were performed on live cells using standard protocols. For measurements involving tagRFP expression or CFL555-labeled antibodies, fluorescence was collected through a 585/40 nm emission filter under excitation at 473 nm and 532 nm to distinguish specific fluorescence from cellular autofluorescence. The two channels (Ex 473 nm/Em 585/40 nm and Ex 532 nm/Em 585/40 nm) were plotted against each other to discriminate true fluorophore-positive events (high 532 nm/473 nm ratio) from autofluorescent populations (lower ratio). When detecting ERBB2 with CFL555-conjugated secondary antibodies, fluorescence intensities on both axes are labeled as *ERBB2 (CFL555)* with corresponding excitation wavelengths. FACS analysis was performed on a FACSVantage SE flow cytometer (Beckton-Dickinson, Franklin Lakes, NJ, USA). Data were further processed and visualized using Cytobank Community Edition (https://community.cytobank.org).

### 2.6. Luciferase Assay

An assay for steady firefly luciferase luminescence (TRIS 100 mM, MgSO_4_ 60 mM, DTT 5 mM, EDTA 4 mM, ATP 4 mM, Luciferin 0.6 mM, Glycerol 10%, CoA 0.4 mM, Na_2_P_2_O_7_ 0.1 mM) was performed in white 96-well plates (SPL Life Sciences, Pocheon-si, Gyeonggi-do, Korea) according to standard protocol. Luminescence readings were collected using a Triad microplate reader (Dynex Technologies, Chantilly, VA, USA).

### 2.7. Alpaca Immunization and VHH Nucleotide Sequences Propagation

Vesicular stomatitis virus was produced in the BHK-21 cell line and purified using the standard ultracentrifugation technique. About 1010 IFU in 200 μL PBS were inactivated by adding 200 nM cm11mYU11 [35] and incubating under fluorescent light for 1 h. Virus preparation was then mixed with an equivalent volume of Freund’s complete adjuvant (Pierce, WA, USA). The mixture was injected into the hind right leg of an alpaca (Vicugna pacos). Two additional boosts were performed with intervals of 3 weeks. Five days after the last boost, 120 mL of venous blood was collected from the external jugular vein. The blood was then separated in a Ficoll gradient, and the PBMC fraction was isolated.

### 2.8. RNA Isolation, Reverse Transcription, and Quantitative PCR

RNA isolation from cells was performed using ExtractRNA reagent (Evrogen, Russia) according to the manufacturer’s recommendations. Purified RNA was used for cDNA synthesis using SuperScript III Reverse Transcriptase (Invitrogen, Waltham, MA, USA). The reaction was carried out according to the manufacturer’s instructions. For specific cDNA synthesis, the primer CH2_IgG_sp rev (5′-GGTACGTGCTGTTGAACTGTTCC-3′) was used. The mixture was incubated for 1 h at 50 °C, followed by 15 min at 70 °C. cDNA preparations were stored at −20 °C until use.

### 2.9. Phage Display

Amplification of VHH fragment coding sequences from cDNA was performed using Deep Vent DNA polymerase (New England Biolabs, Ipswich, MA, USA). The primers used were Alp_VVH3_uni_fwd (5′-GAACAGACCACCATGTCTAGASAGKTGCAGSTSGTRGAGTCTGKGGGAGG-3′), Alp_VHH_R2 (5′-CCTTGTAATCCGGATCCGGTTGTGGTTTTGGTGTCTTGGG-3′), and Alp_VHH_R1 (5′-CCTTGTAATCCGGATCCGGGGGGTCTTCGCTGTGGTGCG-3′). This primer combination inserts XbaI and BamHI restriction sites at the amplicon ends. Amplification was performed under the following thermocycling conditions: 30 cycles of denaturation at 95 °C for 30 s, annealing at 61 °C for 30 s, and elongation at 72 °C for 30 s. Reaction quality was assessed by agarose gel electrophoresis.

Amplicons were isolated from the gel using the CleanUp Mini kit (Evrogen, Russia). Purified DNA preparations were subjected to hydrolysis using XbaI and BamHI enzymes. The hydrolyzed fragments were re-purified and ligated into 500–1000 ng of pHEN2-XB phagemid vector overnight. T4 DNA ligase (Evrogen, Russia) was used as the enzyme. After enzyme inactivation, the ligation mixture was transformed into E. coli strain TG-1 according to a previously described protocol [36]. Following transformation, cells were plated on agar nutrient medium plates and incubated at 30 °C. After colony growth, the library was collected and frozen in 20% glycerol stocks for storage and subsequent phage library production. Bacteriophage production was performed according to a previously described protocol [37].

### 2.10. Selection

For the selection of VSV-G protein-specific variants from the phage library, concentrated live VSV virus was used. For the selection of variants specific to the VSV-G protein from the phage library, concentrated live VSV virus was used. Maxibinding immunological tubes (SPL Lifesciences, Korea) were employed for the phage selection. The live virus was adsorbed overnight at a quantity of 109 IFU per tube for the first selection and 109 for the second. The tubes were blocked for 2 h with 5% MPBS solution at 37 °C. Bacteriophages suspended in MPBS at a concentration of 1 × 10^12^ particles were then applied and incubated with constant mixing for 2 h. Subsequently, the tubes were washed ten times sequentially with PBST and PBS solutions, each wash lasting 30 s with shaking. Phages were eluted by incubating the tubes for 30 min with constant mixing using 1 mL of 100 mM triethylamine solution. To the eluate, 500 μL of 1 M Tris buffer (pH 7.4) was added for neutralization. Then, 750 μL of the neutralized phage solution was added to 9.25 mL of exponential-phase TG-1 culture and incubated for 30 min. The culture was pelleted by centrifugation and plated on nutrient agar plates. Parallel serial dilutions were performed to estimate colony counts. The selection procedure was carried out in two rounds.

Additionally, selection on membranes without the use of immunological tubes was also conducted. Whole-virus electrophoresis was performed in a 10% acrylamide gel, followed by transfer to a PVDF membrane for 4 h at 200 mA. The membrane was then stained for 30 min with 0.1% Ponceau solution in 5% acetic acid. Excess dye was removed by three 5 min washes in distilled water. Based on published data [38], the VSV-G protein location was visually assessed, and the corresponding fragment was excised from the membrane. The excised fragment was blocked for 1 h in 5% MPBS solution, then incubated for 2 h in 3 mL of 5% MPBS solution containing 1 × 10^12^ bacteriophage particles with constant mixing. The membrane was washed six times in 0.05% PBST solution with constant mixing. After washing, the membrane was incubated in 5 mL of TG-1 cell suspension in the logarithmic growth phase. Following infection, cells were plated on agar nutrient medium plates, and serial dilutions were prepared for colony count assessment. The selection procedure was performed twice.

### 2.11. Individual Clone Selection

After the second selection round, 96 clones were seeded in wells of a 96-well plate in LB medium with carbenicillin (100 μg/mL) and grown at 37 °C overnight. The following day, 40 μL from the overnight culture was transferred to 800 μL of LB medium with carbenicillin, grown for 2 h, and infected with M13 bacteriophage. After infection, cultures were grown for an additional hour, kanamycin (50 μg/mL) was added, and growth continued at 37 °C for another 4 h. The plate was centrifuged for 15 min at maximum speed. The supernatant of each well contained bacteriophage from individual clones.

Clone specificity testing was performed using ELISA. On immunological plates, 50 μL of antigen at a 3 μg/mL concentration was sorbed overnight. After incubation, plates were blocked with 5% MPBS solution at 37 °C for 2 h. Control plates were blocked without antigen addition. Following blocking, 100 μL of supernatant containing bacteriophages from individual clones was added to the corresponding wells on each plate. Incubation was performed on a shaker for 2 h. Washing was performed in triplicate with 5 min intervals, first with PBST, then with PBS. HRP-conjugated anti-M13 antibodies (Sino Biological, Beijing, China) were used as secondary antibodies. Staining was performed on a shaker for 40 min, followed by washing. Development was carried out with TMB solution, stopped with 5% sulfuric acid. Results were recorded using a Triad LT microplate analyzer (Dynex Technologies, Chantilly, VA, USA) at 450 nm wavelength.

### 2.12. Protein Production and Purification

Preparative quantities of selected nanobodies were obtained as His-tagged proteins at the C-terminus through expression in E. coli BL21(DE3) BirA culture. For this purpose, corresponding VHH sequences were pre-cloned into the pET32b+ expression vector. VHH fragment expression was performed using the autoinduction method in ZYM-5052 medium. Cells were grown for approximately 24 h at 30 °C with constant mixing, after which the suspension was pelleted by centrifugation at 6000× *g* for 8 min at 4 °C. The resulting pellet was lysed for 30 min with constant mixing in buffer containing 50 mM Tris (pH 7.5), 0.2 mg/mL lysozyme, 300 mM NaCl, 0.1% Triton X-100, 1 mM PMSF, and 10% glycerol. Subsequently, the lysate was sonicated with 10 consecutive 60 s treatments with 30 s intervals. To prevent overheating, the sample was kept on ice during the procedure. Centrifugation was then performed at maximum speed for 4 h at 4 °C to clarify the lysate. Target protein purification was accomplished using affinity chromatography on Ni-NTA resin (Bio-Rad). Supernatants were passed through a column containing 1 mL of resin at 1 mL/min, then the column was sequentially washed with 20 column volumes of 20 mM and 50 mM imidazole solutions, after which the target protein was eluted with 3.5 mL of 500 mM imidazole solution. For additional purification, samples were subjected to gel filtration on a HiPrep Sephacryl S200HR column (Cytiva) using phosphate-buffered saline. Final product purity was assessed by electrophoresis in a 15% SDS-PAGE gel, followed by Coomassie Brilliant Blue staining.

### 2.13. Murine Xenograft Model

The aim of this pilot experiment was to obtain preliminary evidence of in vivo targeting by the 929-B6 adapter molecule. Six female BALB/c mice (4 weeks old, specific pathogen-free) were used as individual experimental units. Mice were housed in groups of three per ventilated cage under standard conditions (22 ± 2 °C, 55 ± 10% humidity, 12 h light/dark cycle) with food and water available ad libitum.

Each mouse was orthotopically implanted into the mammary fat pad with 3 × 10^5^ EMT-ERBB2 cells suspended in 50 μL of PBS. Tumors developed within two weeks. All mice in which palpable or visible tumors formed were included; no exclusion criteria were set, and no animals were excluded.

Mice were randomly assigned using a random-number generator to three groups (*n* = 2 per group):

1. LVs pseudotyped with wild-type VSV-G,
2. LVs pseudotyped with receptor-ablated VSV-G_mut_ (control),
3. VSV-G_mut_-pseudotyped LVs pre-incubated with 1.5 ug/mL 929-B6.

Approximately 10^7^ or 10^8^ infectious units (1 × 10^6^ or 1 × 10^7^ pg of p24 equivalent) of each LV preparation were concentrated with PEG-8000, resuspended in 200 μL PBS, incubated with 929-B6 where indicated, and injected intraperitoneally, once, one animal per dose level. The experimental unit was a single mouse.

Because of the small pilot sample size, potential confounders such as cage location, handling order, and imaging sequence were not formally controlled; all animals were maintained under identical housing and procedural conditions to minimize environmental variation.

Five days later, the mice were anesthetized with a mixture of zoletil (20 mg/kg) and xylazine (5 mg/kg) and injected intraperitoneally with 30 ug furimazine. Bioluminescent images were acquired once using the LumoTrace Fluo (Abisense, Russia) imaging system, and luminescence originating from tumor regions was visually compared between groups. This was an exploratory pilot study rather than a hypothesis-testing experiment; therefore, no a priori sample-size calculation was performed. The primary outcome measure was the detection of tumor-localized bioluminescence, used qualitatively to assess vector targeting. No blinding was applied. Because of the exploratory design and limited sample size, no statistical analyses were performed. Animals were monitored daily, and no adverse events occurred. Orthotopic implantation was used to model physiological tumor growth, and intraperitoneal injection was chosen as a simple systemic route for preliminary evaluation of 929-B6 targeting.

To calculate the on-to-off target ratio, total photon flux values were obtained for two regions of interest (ROIs): the tumor area and the abdominal area. For each ROI, the background signal measured in an adjacent region of equal area was subtracted. The on-to-off ratio was then calculated as:

$$on/off\;ratio=\frac{tumor\;photon\;flux}{\left(\mathrm{abdominal}\;\mathrm{photon}\;\mathrm{flux}\;-\;\mathrm{tumor}\;\mathrm{photon}\;\mathrm{flux}\right)}$$

### 2.14. Statistical Analyses

Statistical comparisons between multiple groups were performed using one-way ANOVA followed by Tukey’s post hoc test. Significance levels are indicated in the figures as follows: *p* < 0.05 (*), *p* < 0.01 (**), *p* < 0.001 (***), and *p* < 0.0001 (****). The statistical analysis was performed using GraphPad Prism 10.

## 3. Results

**Figure 1:**
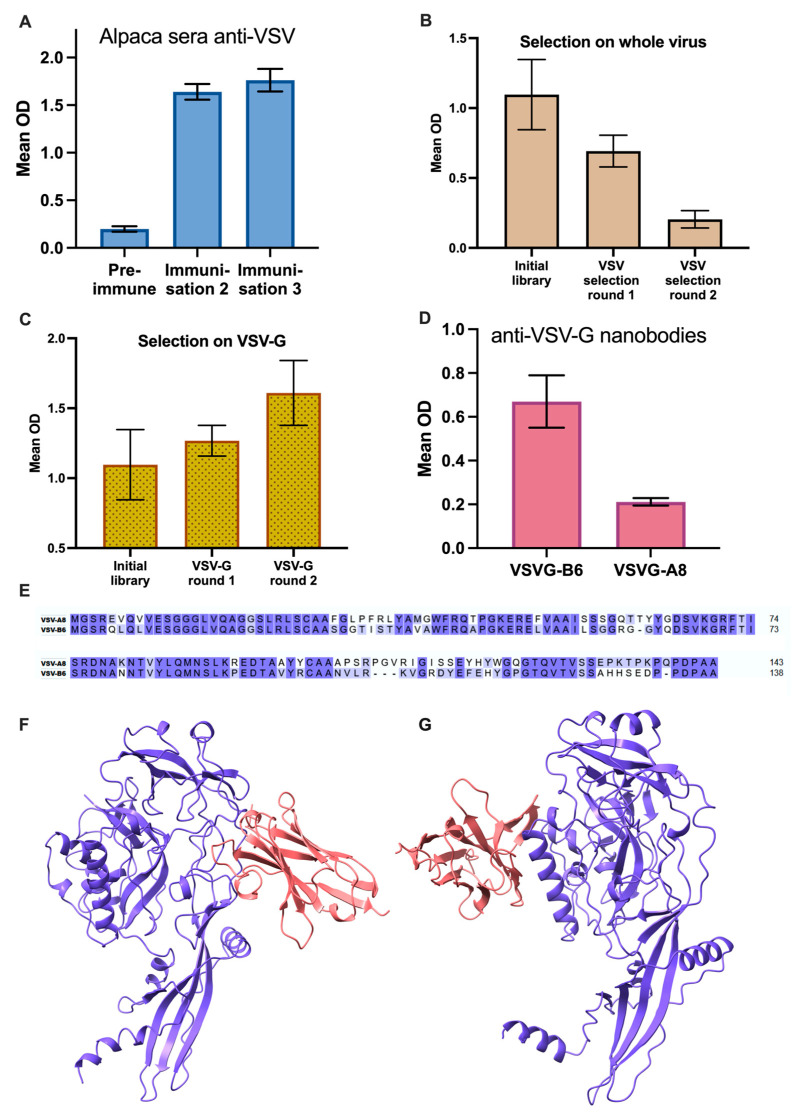
(**A**) Specific activity of pre-immune and post-immunization alpaca sera against VSV virions. (**B**) Anti-VSV reactivity of pooled phage libraries during selection on surface-immobilized VSV particles. (**C**) Anti-VSV reactivity of phage libraries during selection on membrane-bound VSV-G protein. (**D**) Anti-VSV reactivity of VSVG-B6 and VSVG-A8 nanobodies. (**E**) Sequence alignments of VSVG-A8 and VSVG-B6 nanobodies. (**F**,**G**) Models of complex structures of VSVG-A8 (**F**) and VSVG-B6 (**G**) with VSV-G monomer.

To generate the lentiviral particle-binding component of our adapter molecule, we isolated a nanobody specific for the VSV-G glycoprotein. After three rounds of alpaca immunization with inactivated vesicular stomatitis virus, serum antibody titers against VSV markedly increased (Figure 1A). A phage display library comprising >10^7^ independent VHH clones was constructed from peripheral B-cell cDNA. Two initial selection rounds on immobilized intact virions did not enrich VSV-binding clones, as ELISA signals decreased relative to the primary library (Figure 1B), suggesting that whole-virus presentation was suboptimal for isolating VSV-G–specific binders.

To focus selection on the envelope protein itself, viral proteins were resolved by SDS-PAGE, transferred to PVDF membrane, and the band corresponding to VSV-G was excised and used as an antigen in two additional selection rounds. Under these conditions, strong enrichment of VSV-reactive phages was observed (Figure 1C). Screening of individual clones identified two unique nanobody sequences, designated VSVG-A8 and VSVG-B6 (Figure 1E). Both bound purified VSV-G in ELISA (Figure 1D). Structural modeling using AlphaFold 3 indicated distinct binding regions: VSVG-A8 near segments S2–S3 and VSVG-B6 at the trimerization domain (Figure 1F,G). Based on higher binding activity, VSVG-B6 was selected as the VSV-G-binding component of the bispecific adapter.

**Figure 2:**
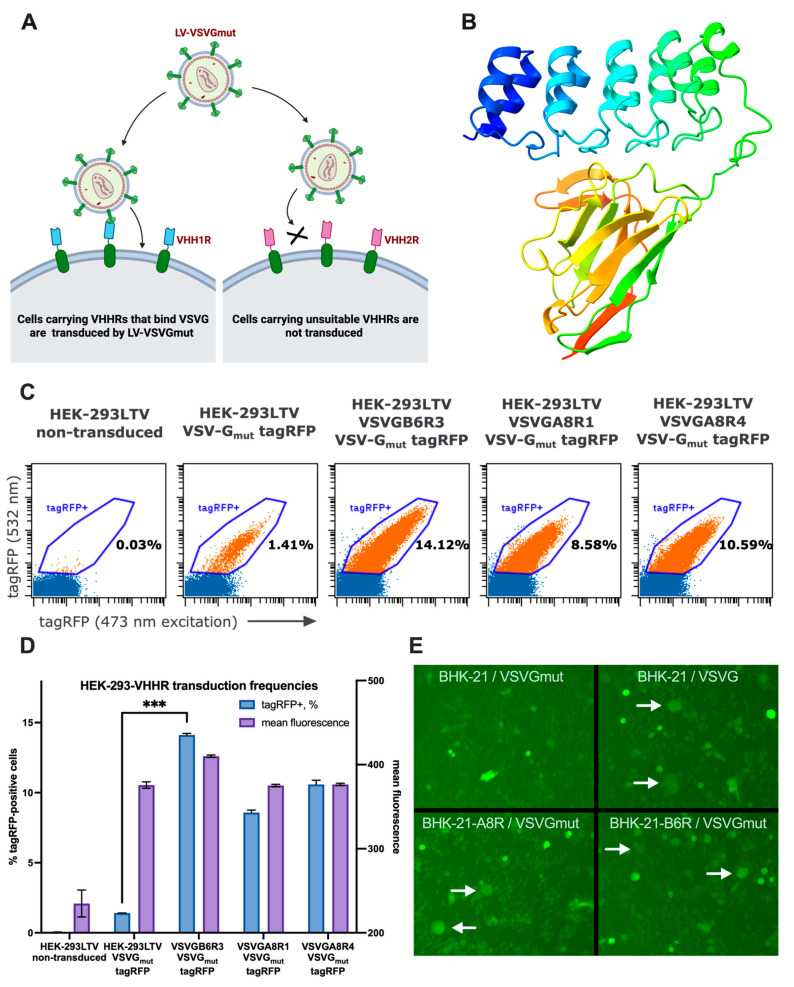
(**A**) Schematic representation of the VHH-pseudoreceptor assay. (**B**) Structural model of 929-B6 adapter protein. (**C**) Flow cytometric analysis of VHH-pseudoreceptor-assisted (VSVGB6R3, VSVGA8R1, VSVGA8R4) and unassisted (HEK-293LTV) transduction by VSV-G_mut_-pseudotyped lentiviral vectors carrying the tagRFP expression cassette. (**D**) Transduction frequencies and mean fluorescence intensities of tagRFP-positive populations. (**E**) Syncytia formed on HEK-293LTV-VSVGB6R3, HEK-293LTV-VSVGA8R1, HEK-293LTV-VSVGA8R4, and HEK-293LTV following transduction with pMD2G_mut_ and pLCMV-tagRFP. ***—*p* = 0.0002.

To evaluate whether VSVG-A8 or VSVG-B6 can mediate targeted transduction of VSV-G-pseudotyped lentiviral particles, we expressed these nanobodies on the surface of HEK-293LTV cells (a subclone of the HEK-293T line) as pseudoreceptors (Figure 2A). The resulting cell lines, HEK-293LTV VSVGB6R3, HEK-293LTV VSVGA8R1, and HEK-293LTV VSVGA8R4, along with the parental HEK-293LTV cells lacking pseudoreceptor expression, were transduced with LVs carrying a tagRFP expression cassette and pseudotyped with VSV-G_mut_. Flow cytometric analysis of fluorescent cell populations (Figure 2C) revealed that the transduction efficiencies of the pseudoreceptor-expressing lines were 6–10-fold higher than those of the parental cells (Figure 2D). This result indicates that the presence of anti-VSV-G pseudoreceptors compensates for the reduced affinity of VSV-G_mut_ toward LDLR. As an additional assay to validate receptor–ligand interactions, we performed transient transfections of BHK-21 cells expressing VSVGB6R or VSVGA8R with a mixture of plasmids encoding VSV-G_mut_ (pMD2.G_mut_) and tagGFP2 (pLCMV-tagGFP2). In both BHK-21-VSVGB6R and BHK-21-VSVGA8R cells, we observed the formation of small syncytia, indicative of membrane fusion activity mediated by VSV-G_mut_ binding to its respective pseudoreceptor. In contrast, control BHK-21 cells transfected with pMD2.G_mut_ alone exhibited no syncytium formation (Figure 2E). Because VSVG-B6 exhibited slightly stronger binding activity than VSVG-A8 (Figure 1D), it was selected as the VSV-G-binding component of the adapter molecule. By fusing the coding sequence of the anti-ERBB2 DARPin 9.29 to that of VSVG-B6 via a flexible linker, we generated the bispecific adapter molecule 929-B6 (Figure 2B).

**Figure 3:**
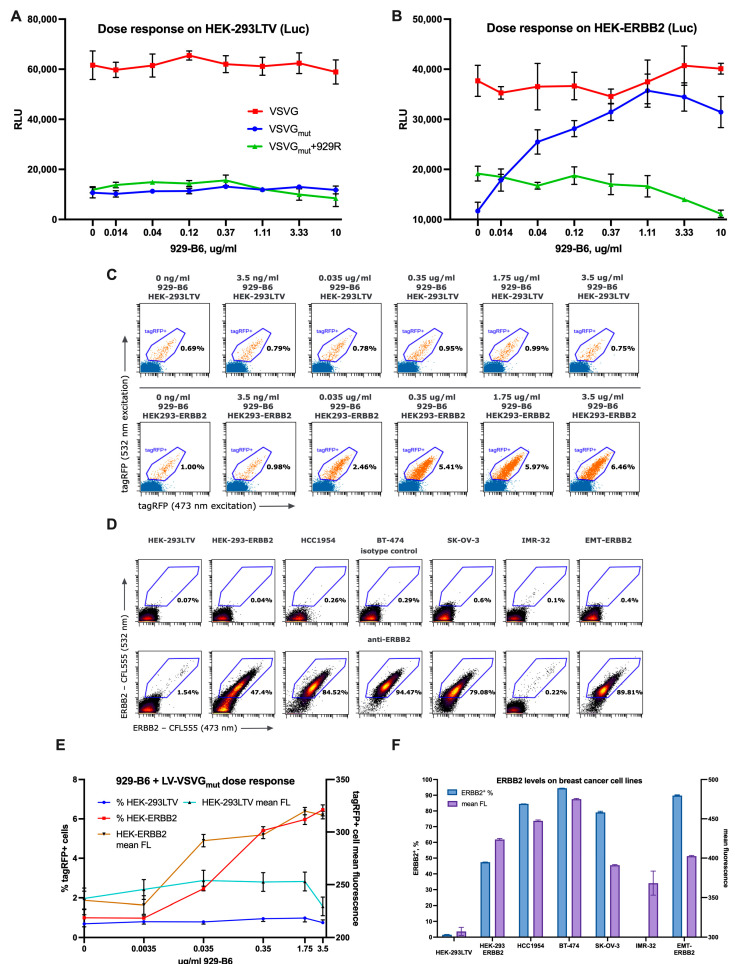
(**A**) Dose response curves measured on HEK-293LTV cells transduced with VSV-G, VSV-G_mut_, and VSV-G_mut_ + 929R-pseudotyped LVs carrying the firefly luciferase expression cassette and pre-treated with 929-B6 in marked concentrations. (**B**) Dose–response curves measured on HEK-ERBB2 cells transduced with the same LV pseudotypes and in the same conditions as above. (**C**) Flow plots of the 929-B6 dose–response test. (**D**) Flow plots of a panel of anti-ERBB2-stained cell lines. (**E**) Dose–response curves and mean fluorescence intensities of tagRFP+ populations measured in HEK-293LTV and HEK-ERBB2 cells transduced with VSV-G and VSV-G_mut_-pseudotyped LVs carrying the tagRFP expression cassette and pre-treated with 929-B6 in marked concentrations. (**F**) Percent values of ERBB2-positive cells and corresponding mean fluorescence intensities measured on a panel of cell lines.

To evaluate the targeting activity of 929-B6, we generated a derivative of the HEK-293LTV cell line, designated HEK-ERBB2, by introducing a lentiviral construct encoding human ERBB2, followed by selection for stable expression. Flow cytometric analysis confirmed that approximately 50% of the cells displayed high levels of ERBB2 on their surface (Figure 3F). We then transduced parental HEK-293LTV and HEK-ERBB2 cells with VSV-G_mut_-pseudotyped LVs carrying a firefly luciferase reporter in the presence of varying concentrations of 929-B6 and quantified transduction efficiency by measuring luciferase activity. For comparison, we included VSV-G_mut_-pseudotyped LVs used together with the 929 pseudoreceptor (VSVGm + 929R), representing the conventional pseudoreceptor-based targeting approach.

The results (Figure 3A–C) showed that the addition of 929-B6 to LVs transducing ERBB2-negative HEK-293LTV cells did not significantly affect transduction efficiency, although a modest reduction in titers was observed at higher 929-B6 concentrations. In contrast, 929-B6 markedly enhanced transduction of HEK-ERBB2 cells by VSV-G_mut_-pseudotyped LVs, with maximal luciferase activity observed at approximately 1.1 µg/mL. Further increases in 929-B6 concentration led to a gradual decline in signal, a trend also observed for VSVGm + 929R LVs. The infectivity of unmodified VSV-G-pseudotyped LVs remained largely unaffected by 929-B6 addition.

To validate these findings, we conducted independent transductions of HEK-293LTV and HEK-ERBB2 cells with VSV-G_mut_-pseudotyped LVs carrying the tagRFP expressor, preincubated with 929-B6. Consistent with luciferase data, the highest proportion of highly fluorescent cells was detected in samples treated with 1.75 µg/mL 929-B6, closely matching the optimal range identified in the previous experiment (Figure 3C,E). At higher concentrations, mean fluorescence intensity decreased slightly, while the overall percentage of transduced cells remained largely unchanged.

We next evaluated the performance of VSV-G_mut_-pseudotyped lentiviral vectors preincubated with 929-B6 across three breast cancer cell lines with high ERBB2 expression and one ERBB2-negative control line. Surface expression of ERBB2 was verified by staining with an anti-ERBB2 antibody. Among the analyzed lines, BT-474 exhibited the highest expression levels, followed by HCC1954, whereas SKOV3 cells showed the lowest ERBB2 expression both in terms of the proportion of positive cells and mean fluorescence intensity (Figure 3D,F). The ERBB2-positive fraction in the IMR-32 cell line was negligible.

**Figure 4:**
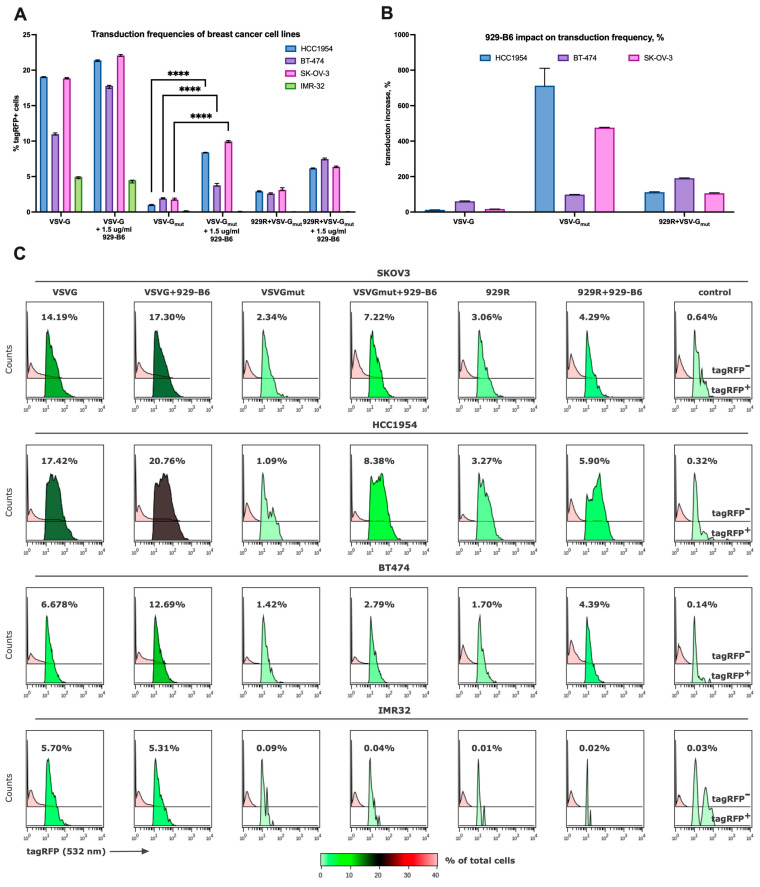
(**A**) Transduction frequencies of ERBB2-positive breast cancer cell lines and control ERBB2-negative cell line IMR32. (**B**) 929-B6 impact (fold increase in transduction frequencies) on transduction efficiency of ERBB2-positive breast cancer cell lines. (**C**) Flow plots of cell line panel transduced with VSV-G, VSV-G_mut_, and VSV-G_mut_ + 929R-pseudotyped LVs with and without 929-B6 pre-treatment. ****—*p* < 0.0001.

To benchmark our approach, we additionally performed transductions using VSV-G_mut_ + 929R-pseudotyped LVs. The results indicated that the intrinsic susceptibility of a given cell line to lentiviral transduction remained a key determinant of overall efficiency. SKOV3 cells demonstrated the highest transduction efficiency, closely followed by HCC1954 and BT-474, while IMR-32 cells were minimally transduced (Figure 4A). The strongest reporter expression levels were observed in HCC1954 cells, followed by SKOV3 and BT-474 (Figure 4C and Figure S1).

Pre-treatment of LVs with 1.5 µg/mL 929-B6 enhanced transduction yields for all pseudotypes tested. The effect was least pronounced for native VSV-G-pseudotyped LVs, moderate for VSV-G_mut_ + 929R, and most substantial, up to an eight-fold increase, for VSV-G_mut_-pseudotyped LVs (Figure 4B). In contrast, the addition of 929-B6 did not significantly affect the transduction of ERBB2-negative IMR-32 cells. For VSV-G_mut_-pseudotyped LVs, the magnitude of the 929-B6 effect correlated with the mean fluorescence intensity of transduced populations, suggesting that in cell lines exhibiting lower transgene expression, the true enhancement may be underestimated due to overlap between tagRFP-positive and tagRFP-negative fractions.

To further evaluate the selectivity of 929-B6-mediated targeting, we tested VSV-G-, VSV-G_mut_-, and VSV-G_mut_ + 929R-pseudotyped lentiviral vectors on mixed populations of ERBB2-positive and ERBB2-negative cells. Because intrinsic differences in cell line susceptibility can strongly affect transduction efficiency, we employed a single cell line system consisting of HEK-293LTV and HEK-ERBB2. Mixed cultures containing 10%, 23%, 30%, or 45% ERBB2^+^ cells were transduced with each LV pseudotype, with or without 929-B6 pre-treatment, and analyzed for reporter expression within ERBB2^+^ and ERBB2^−^ subpopulations.

**Figure 5:**
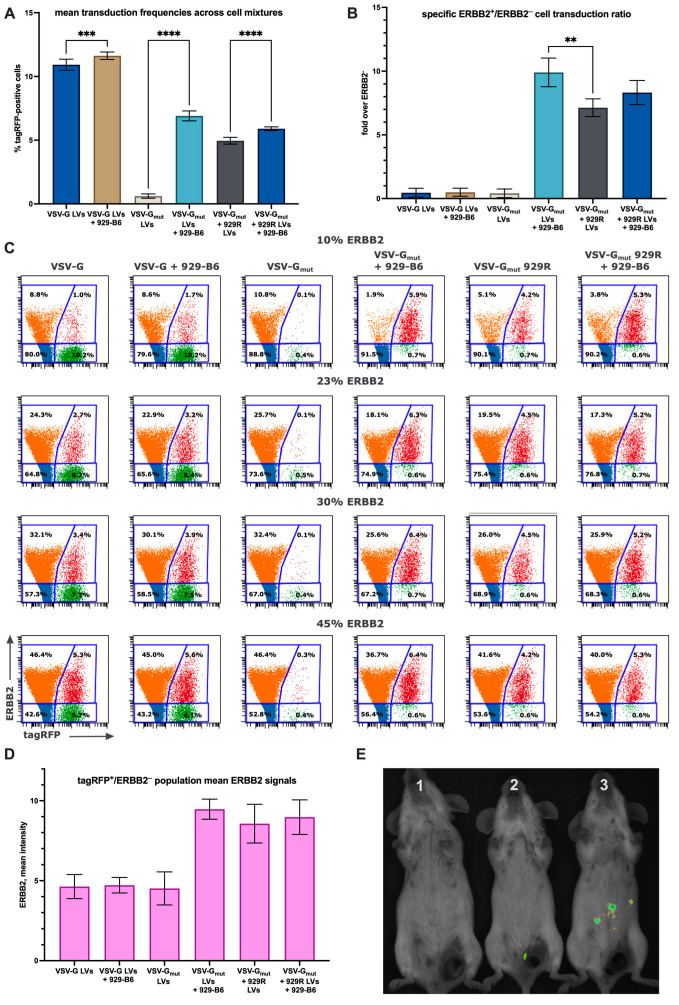
(**A**) Comparison of mean transduction titers of 929-B6 pre-treated and untreated LVs measured across four mixtures of ERBB2-negative and ERBB2-positive cells. (**B**) Specific (ERBB2^+^/ERBB2^−^) transduction ratios of corresponding LV pseudotypes and 929-B6 treatment statuses. (**C**) Flow plots of cell mixtures transduced with tagRFP-expressing LVs with and without 929-B6 treatment, stained with anti-ERBB2 antibody. Blue: ERBB^−^/tagRFP^−^, orange: ERBB^+^/tagRFP^−^, green: ERBB^−^/tagRFP^+^, red: ERBB^+^/tagRFP^+^. (**D**) Mean fluorescence intensities (ERBB2) of ERBB2^−^/tagRFP^+^ subpopulations from the above plots. (**E**) Bioluminescence imaging of EMT-ERBB2-tumor-bearing mice injected with 10^8^ i.f.u. of VSV-G_mut_ (1), VSV-G_mut_ + 929-B6 (2), and VSV-G-pseudotyped (3) LVs carrying the nanoLuc expression cassette. Comparisons were performed with one-way ANOVA, **—*p* = 0.0062, ***—*p* = 0.0007, ****—*p* < 0.0001.

The results confirmed that pre-incubation with 929-B6 substantially enhanced overall transduction efficiency across all pseudotypes and ERBB2^+^/ERBB2^−^ ratios (Figure 4A), while preferentially increasing transduction of ERBB2-positive cells (Figure 4C). For VSV-G_mut_ pseudotypes treated with 929-B6, as well as for VSV-G_mut_ + 929R and VSV-G_mut_ + 929R + 929-B6, no distinct ERBB2^−^/tagRFP^+^ population was detected, and mean fluorescence intensity within the ERBB2^−^/tagRFP^+^ gate shifted markedly upward compared with untreated VSV-G_mut_ samples (Figure 5D). Because the effective infectious titers of VSV-G_mut_ and VSV-G_mut_ + 929R LVs represented less than 10% of total cell counts, we did not observe a measurable decrease in tagRFP-positive cell frequency in samples containing lower fractions of ERBB2^+^ cells. Comparison of selectivity ratios revealed that VSV-G_mut_ LVs pre-incubated with 929-B6 achieved significantly higher ERBB2-specific transduction than VSV-G_mut_ + 929R LVs without 929-B6 pre-treatment.

To assess 929-B6-mediated targeting in vivo, we conducted a pilot experiment in mice bearing ERBB2-positive tumor xenografts. The previously described murine EMT-ERBB2 cell line was orthotopically implanted into syngeneic mice, which were subsequently injected intraperitoneally with nanoLuc-expressing LVs pseudotyped with either VSV-G or VSV-G_mut_, with or without 929-B6 pre-treatment. Following administration of the nanoLuc substrate furimazine, bioluminescent imaging was performed. Low luminescence levels were detected in two mice receiving VSV-G-pseudotyped LVs, whereas a weak, tumor-localized signal was observed in one mouse treated with VSV-G_mut_ + 929-B6 (Figure 5E). No tumor-associated luminescence was detected in animals injected with LVs lacking 929-B6 pre-treatment.

## 4. Discussion

The potential application of lentiviral vectors (LVs) in cancer therapy often requires targeted delivery of therapeutic payloads to tumor cell populations expressing distinct surface receptors (tumor-associated antigens) [39]. Conventional approaches, such as pseudotyping LVs with receptor-ablated glycoproteins fused to targeting domains or utilizing pseudoreceptor-mediated retargeting, necessitate the generation of a separate pseudotype for each receptor of interest [40]. Because LV production is both costly and time-consuming, we sought to develop a strategy that would enable rapid retargeting of a single, unified LV stock through the use of an auxiliary protein adapter.

Nanobodies, the antigen-binding domains of camelid heavy-chain-only antibodies, represent an ideal foundation for such adapters due to their compact size, high stability, and ease of selection from immune libraries [41,42]. We therefore immunized an alpaca with whole inactivated vesicular stomatitis virus (VSV) and carried out phage-display selections, first using intact viral particles, and subsequently, after limited enrichment, using purified VSV-G immobilized on PVDF membranes, which allowed us to concentrate the antigenic portion while reducing background.

Following identification of VSV-G-reactive nanobody clones, we evaluated their functionality using a reverse-pseudoreceptor assay; nanobodies capable of mediating viral entry under these conditions were considered promising candidates for incorporation into LV retargeting adapters. As an alternative screening strategy, we exploited the intrinsic fusogenic activity of VSV-G. Wild-type VSV-G induces the formation of small syncytia among cells expressing the glycoprotein [43], whereas receptor-ablated VSV-G_mut_ are expected to show strongly reduced ability to promote cell–cell fusion. We hypothesized that restoration of this fusogenic activity through nanobody-mediated pseudoreceptor engagement could serve as an indicator of functional interaction. Although this approach confirmed previously identified VSV-G_mut_-binding nanobodies, it did not yield quantitative data.

The receptor tyrosine-kinase ERBB2 is among the most intensively studied tumor-associated antigens for targeted therapies and thus serves as a highly relevant model system for retargeting lentiviral vectors. Overexpression of ERBB2 occurs in approximately 15–30% of breast cancers and is associated with aggressive disease and poor prognosis [44]. The designed ankyrin repeat protein (DARPin) 9.29, which binds to the membrane-proximal domain IV of ERBB2, is well characterized, with a binding site that partially overlaps that of trastuzumab [32]. Binding near the cell membrane is advantageous, as it allows a 9.29-based adapter molecule to position the lentiviral particle closer to the membrane surface, thereby increasing the likelihood of successful membrane fusion. These properties of DARPin 9.29 were previously exploited for retargeting LVs with measles glycoproteins [12].

The resulting adapter construct, 929-B6, efficiently mediated the entry of lentiviral vectors pseudotyped with either receptor-ablated VSV-G_mut_ or wild-type VSV-G, producing high, receptor-specific infectious titers on ERBB2-positive cells. This effect was further confirmed across a panel of cancer cell lines overexpressing ERBB2. In terms of targeting specificity, the data indicate that the greatest increase in infectious titer between 929-B6-treated and untreated samples correlates with the magnitude of transgene expression. This suggests that the measured specificity values may be underestimated due to partial overlap between transgene-positive and -negative cell populations.

Dose–response experiments revealed that pre-treatment of lentiviral vectors with the 929-B6 adapter at concentrations between 1 and 2 µg/mL resulted in the highest receptor-specific infectious titers. At higher concentrations, a modest decrease in infectivity was observed. We hypothesize that at elevated levels, 929-B6 saturates VSV-G or VSV-G_mut_ molecules on the viral surface, potentially interfering with their fusogenic activity. In another study reporting adapter-mediated retargeting of lentiviral vectors pseudotyped with the Sindbis E2 glycoprotein, the optimal adapter concentration was found to be approximately 50 nM, which also yielded the highest specific infectivity titer [30]. This observation is consistent with our results, where an adapter concentration of 1–2 µg/mL (equivalent to 26–52 nM) produced the best balance between specificity and efficiency.

Studies performed on both the cancer cell panel and mixed ERBB2^+^/ERBB2^−^ cultures demonstrated that pre-treatment with 929-B6 enhanced ERBB2-specific infectivity for lentiviral vectors pseudotyped with either VSV-G or VSV-G_mut_ + 929R. For the VSV-G pseudotype, this effect can likely be attributed to the introduction of additional ERBB2-binding sites on the target cell surface, thereby increasing the probability of successful transduction. In the case of VSV-G_mut_ + 929R pseudotypes, which already utilize ERBB2 for entry, the observed enhancement may indicate that the VSV-G_mut_-to-929R expression ratio used during packaging was suboptimal; additional ERBB2-binding sites provided by 929-B6 could therefore improve overall infectivity. This aligns well with the previous study, where higher concentrations of 9.29 pseudoreceptor-encoding plasmid relative to measles H glycoprotein lead to better specific titers [23].

An additional factor that may contribute to this effect is the uneven distribution of VSV-G_mut_ and 929R molecules on the surface of packaging cells during vector assembly. In wild-type vesicular stomatitis virus, VSV-G accumulates at plasma membrane regions that serve as viral budding sites. VSV-G is inherently organized into membrane microdomains with diameters around 100–150 nm, even in cells expressing G in isolation [45]. HIV-1 Gag multimerization at the inner leaflet defines the budding locus, which leads to VSV-G redistribution to that site and subsequent VSV-G incorporation into LV particles [46]. Consequently, a larger fraction of membrane-associated VSV-G_mut_ is likely incorporated into nascent virions, whereas 929R, being more uniformly distributed across the cell surface, is underrepresented on the viral envelope. This imbalance could account for the suboptimal VSV-G_mut_/929R ratio and explain why additional ERBB2-binding sites provided by 929-B6 enhance infectivity. Taken together, these findings indicate that adapter-based retargeting with molecules such as 929-B6 may offer greater efficiency and flexibility than pseudoreceptor-dependent approaches. When comparing the infectious titers and targeting specificity of 929-B6-retargeted LVs with those of Sindbis-pseudotyped LVs employing modular DARPin 9.29-based targeting [29], our system exhibited substantially higher overall infectivity, albeit with slightly reduced specificity. This difference may reflect the residual capacity of VSV-G_mut_ to interact with other members of the LDL receptor family.

Our preliminary attempt to assess the in vivo performance of VSV-G_mut_ + 929-B6-pseudotyped lentiviral vectors yielded a weak luminescent signal originating from the tumor region. This limited outcome is likely attributable to an insufficient viral dose, a suboptimal route of administration, or inherently low susceptibility of the murine EMT-ERBB2 cell line to lentiviral transduction. Another contributing factor may have been the limited bioavailability of furimazine, which likely reduced the intensity of the luminescent reporter signal [47]. Nevertheless, a weak but detectable luminescent signal originating from tumor tissue was observed following administration of VSV-G_mut_ + 929-B6-pseudotyped vectors, indicating that this targeting approach retains promise for in vivo applications involving therapeutic payloads.

While the 929-B6 construct establishes proof of principle for adapter-mediated retargeting using VSVG_mut_-pseudotyped LVs, this strategy depends on the availability of high-affinity receptor-binding modules that can be fused to the VSV-binding nanobody. At present, a moderate but growing set of such binders exists. These include DARPins, nanobodies, and scFv fragments specific for clinically relevant receptors such as EGFR [48], CD133 [49], CD47 [36], CD30 [50], P-gp [51], EpCAM [9], and others, recently reviewed in [2]. These domains have already been validated in retargeted measles, Nipah, or Sindbis glycoprotein systems, which suggests that their fusion to the VSV-binding moiety could yield functional adapters. Expanding the repertoire of receptor-targeting modules, through DARPin, nanobody, or peptide library selections, will be essential for translating to a wider range of cell types.

The principal advantage of our targeting strategy lies in its capacity to employ a single lentiviral vector stock to generate particles retargeted toward virtually any receptor of interest. Such adaptability could streamline the clinical translation of LV-based therapeutics tailored to diverse cancer phenotypes. Moreover, the finding that the 929-B6 adapter enhances targeted delivery even for wild-type VSV-G-pseudotyped vectors suggests a broader utility of adapter-mediated retargeting. Specifically, this concept could be extended to improve the pharmacological profile of oncolytic vesicular stomatitis virus (oVSV). By conferring additional tropism toward cancer cells, adapter molecules may reduce off-target infection rates and effective dosing requirements, minimize adverse effects, and expand the therapeutic potential of this class of anticancer agents.

## 5. Conclusions

We developed a modular, bispecific adapter 929-B6 that bridges VSV-G, including the LDLR-blind VSV-G_mut_ and a tumor-associated antigen, ERBB2, enabling rapid, switchable retargeting of lentiviral vectors without re-engineering the envelope or co-packaging a pseudoreceptor plasmid. 929-B6 enhanced transduction selectively on ERBB2^+^ cells across multiple assay formats, and improved infectivity for both VSV-G_mut_-pseudotyped vectors and, to a lesser extent, for VSV-G and VSV-G_mut_ + 929R pseudotypes, consistent with a mechanism in which additional target-receptor engagement increases productive entry. Dose–response testing identified a practical operating window (~1–2 µg/mL) above which modest reductions in titer are observed, likely reflecting partial interference with fusogenic steps when virion surfaces become saturated. Compared with pseudoreceptor-dependent retargeting, the adapter strategy reduces production complexity and may alleviate stoichiometric constraints that limit incorporation of targeting moieties into budding particles. Because the VSV-binding and receptor-binding modules are separable, 929-B6 exemplifies a generalizable “plug-in” format that should translate to other receptors by swapping the targeting domain. More broadly, adapter-mediated retargeting may extend to oncolytic VSV and other enveloped vectors, potentially improving tumor selectivity, reducing effective doses, and expanding the therapeutic window for cancer gene therapy.

## Data Availability

The data presented in this study are available in this manuscript.

## Supplementary Materials

The following supporting information can be downloaded at: https://www.mdpi.com/article/10.3390/v17121563/s1, Figure S1: A. Mean fluorescence intensities (MFI) of TagRFP^+^ populations in cancer cell lines transduced with VSV-G, VSV-Gmut and VSV-Gmut+929R-pseudotyped LVs with or without 929-B6 pretreatment. B. MFIs of ERBB^−^/TagRFP^−^, ERBB^+^/TagRFP^−^, ERBB^−^/TagRFP^+^, and ERBB^+^/TagRFP^+^ populations from Figure 5C. C. Control samples corresponding to Figure 5C, compensated. D. Control samples corresponding to Figure 5C, uncompensated. E. Uncompensated flow cytometry plots as in Figure 5C.; Figure S2: A. ELISA p24 quantitation of lentiviral samples. Trendlines were fitted within the 0.5-4 µL range. B. ELISA standard curve for the control p24 sample. C. Calculated p24 concentrations in the tested lentiviral samples.; Figure S3: Quantification of photon flux in bioluminescent imaging of mice receiving 1*108 i.f.u. of LVs.; Figure S4: Quantification of photon flux in bioluminescent imaging of mice receiving 1*107 i.f.u. of LVs.; Figure S5: Transduction frequencies of cancer cell lines relative to ERBB2 expression levels (as transcripts per million, according to CCLE 25q3 data).; Figure S6: Flow plots of cell line panel transduced with VSV-G, VSV-Gmut and VSV-Gmut+929R-pseudotyped LVs with and without 929-B6 pretreatment, as in Figure 4C.
