# Supplementary material for: Switchable Retargeting of Lentiviral Vectors Through a VSV-G-Binding Adapter Molecule
Source: Viruses. 2025 Nov 29;17(12):1563. doi: 10.3390/v17121563 (PMC12737384; doi:10.3390/v17121563)
Supplement: Supplementary file 1 [file viruses-17-01563-s001.zip › viruses-3968451-supplementary.pdf]

Supplementary figures

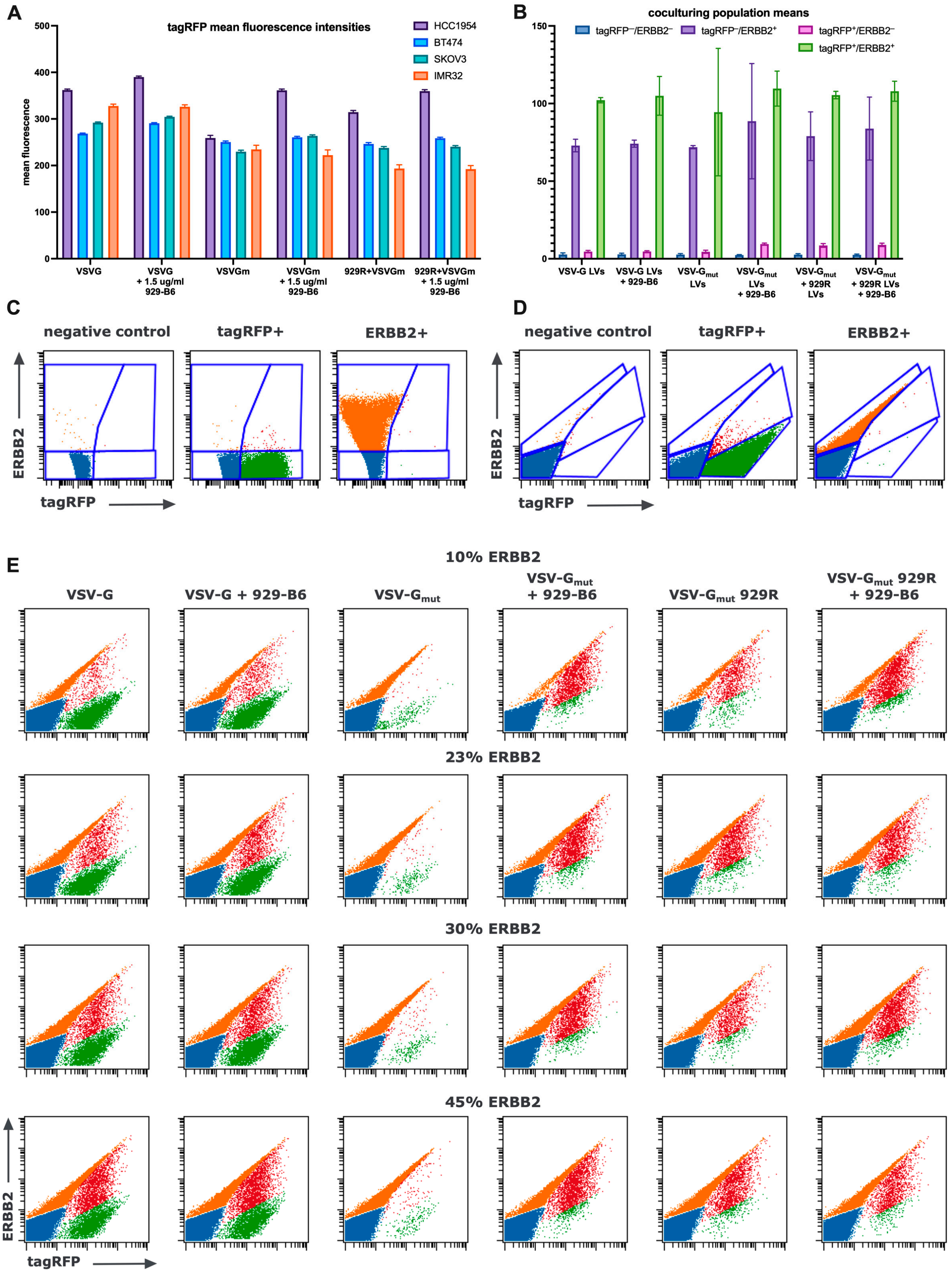


Figure S1. A. Mean fluorescence intensities (MFI) of TagRFP$^+$ populations in cancer cell lines transduced with VSV-G, VSV-G$_{mut}$ and VSV-G$_{mut}$+929R-pseudotyped LVs with or without 929-B6 pretreatment. B. MFIs of ERBB$^-$/TagRFP$^-$, ERBB$^+$/TagRFP$^-$,

ERBB$^-$/TagRFP$^+$, and ERBB$^+$/TagRFP$^+$ populations from Figure 5C. C. Control samples corresponding to Figure 5C, compensated. D. Control samples corresponding to Figure 5C, uncompensated. E. Uncompensated flow cytometry plots as in Figure 5C.

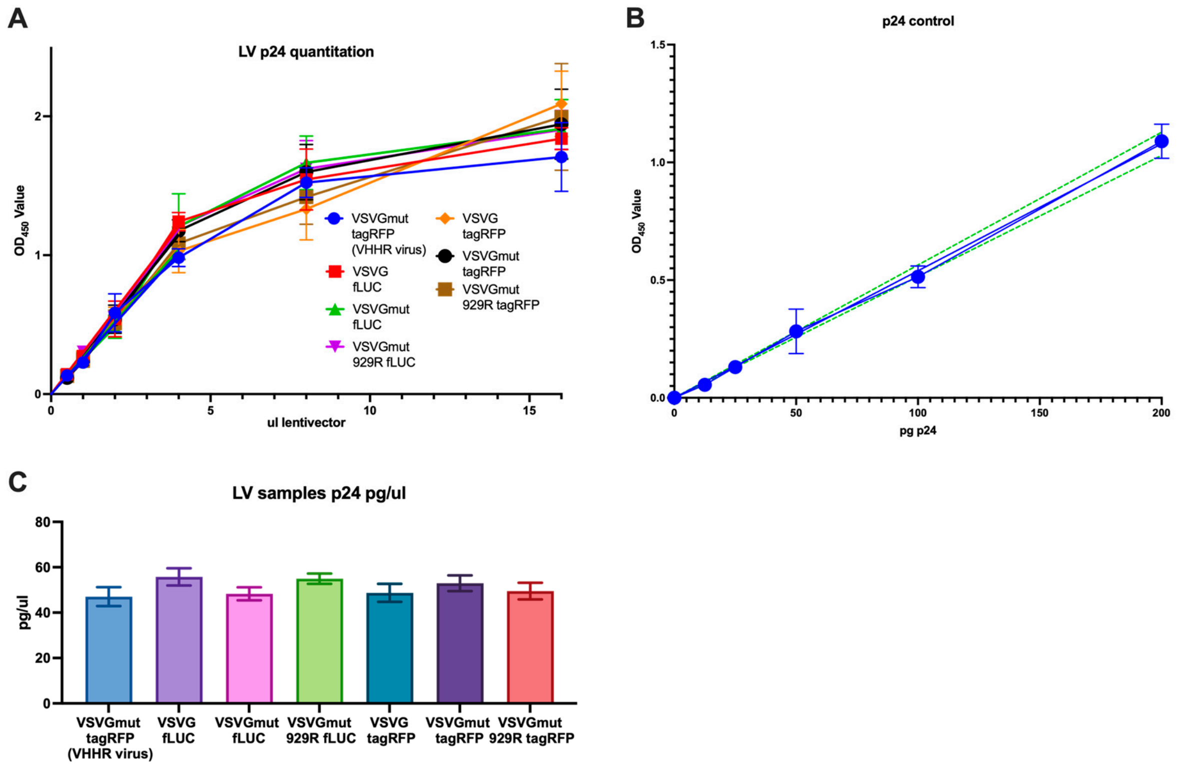


Figure S2. A. ELISA p24 quantitation of lentiviral samples. Trendlines were fitted within the 0.5-4 µL range. B. ELISA standard curve for the control p24 sample. C. Calculated p24 concentrations in the tested lentiviral samples.

A

B

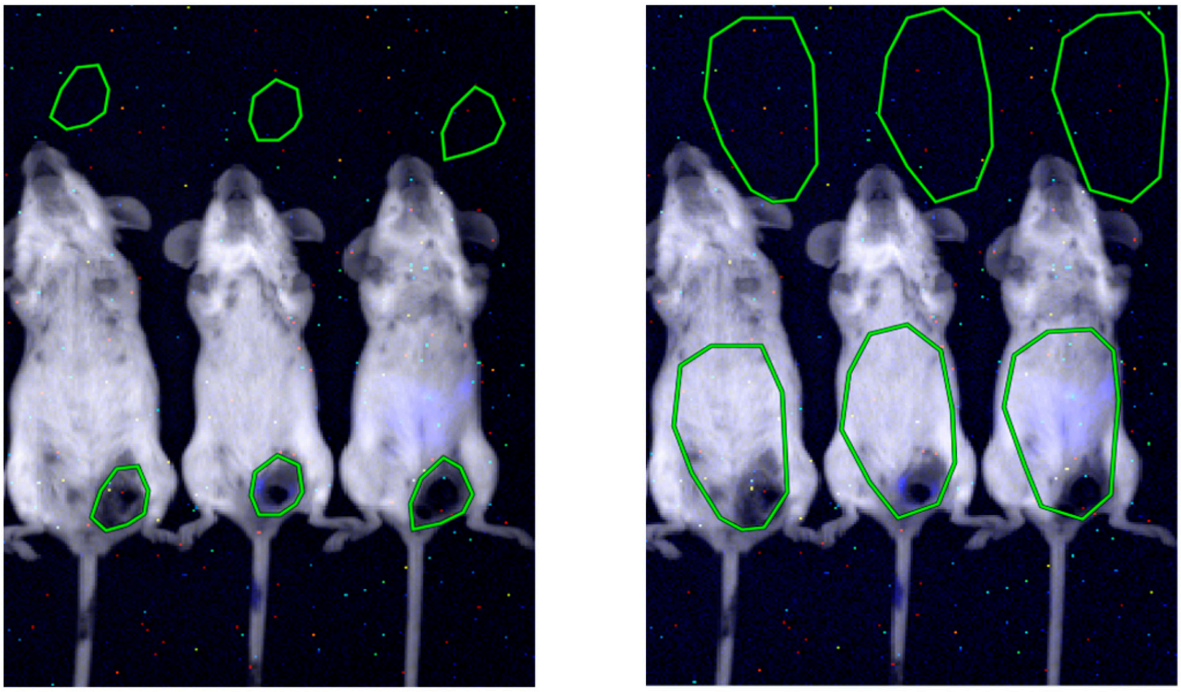

C

| | Radiance (tumor) | Radiance (control region) | Tumor radiance corrected | Radiance (abdomen) | Radiance (control region) | Abdomen radiance corrected | on-to-off ratio |
|---|---|---|---|---|---|---|---|
| VSV-$G_{mut}$ | 2.52e+008 | 2.49e+008 | 3610000.00 | 1.11e+009 | 1.08e+009 | 2.93e+007 | 0.14087572 |
| VSV-$G_{mut}$ + 929-B6 | 2.33e+008 | 2.21e+008 | 1.14e+007 | 1.08e+009 | 1.06e+009 | 1.21e+007 | 16.27294400 |
| VSV-G LVs | 3.75e+008 | 3.37e+008 | 3.84e+007 | 1.02e+009 | 9.64e+008 | 5.30e+007 | 2.62434525 |

Figure S3. Quantification of photon flux in bioluminescent imaging of mice receiving $1*10^8$ i.f.u. of LVs. A. Tumor region of interest (ROI). B. Abdomen ROI. C. Radiance values for each ROI, with calculated corrected radiance for tumor and abdomen, and on/off ratios.

A

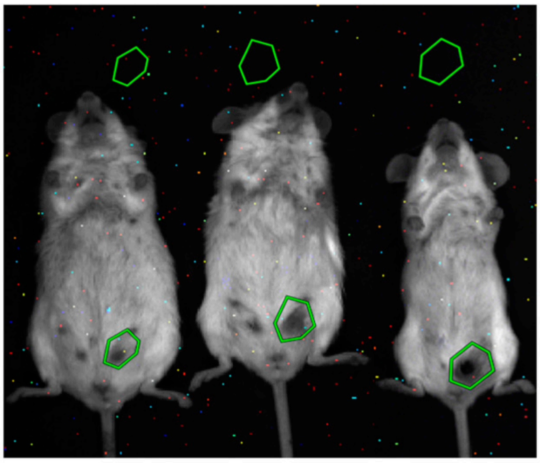

B

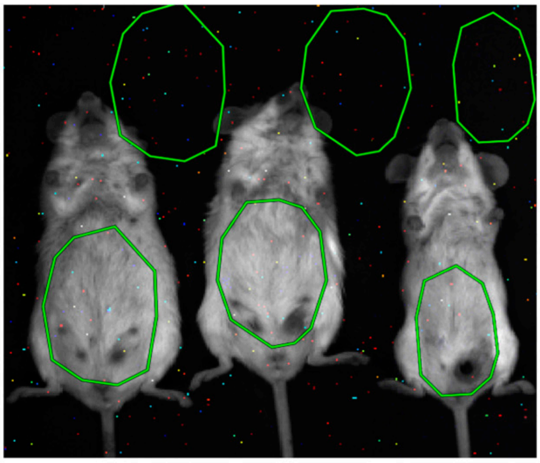

C

| | Radiance (tumor) | Radiance (control region) | Tumor radiance corrected | Radiance (abdomen) | Radiance (control region) | Abdomen radiance corrected | on-to-off ratio |
|---|---|---|---|---|---|---|---|
| VSV-$G_{mut}$ | 1.00e+008 | 9.90e+007 | 1500000.00 | 1.63e+009 | 1.61e+009 | 2.79e+007 | 0.05692851 |
| VSV-$G_{mut}$ + 929-B6 | 1.43e+008 | 1.41e+008 | 1710000.00 | 1.47e+009 | 1.45e+009 | 2.01e+007 | 0.09291774 |
| VSV-G LVs | 1.42e+008 | 1.40e+008 | 1260000.00 | 8.96e+008 | 8.66e+008 | 3.08e+007 | 0.04258315 |

Figure S4. Quantification of photon flux in bioluminescent imaging of mice receiving $1*10^7$ i.f.u. of LVs. A. Tumor region of interest (ROI). B. Abdomen ROI. C. Radiance values for each ROI, with calculated corrected radiance for tumor and abdomen, and on/off ratios.

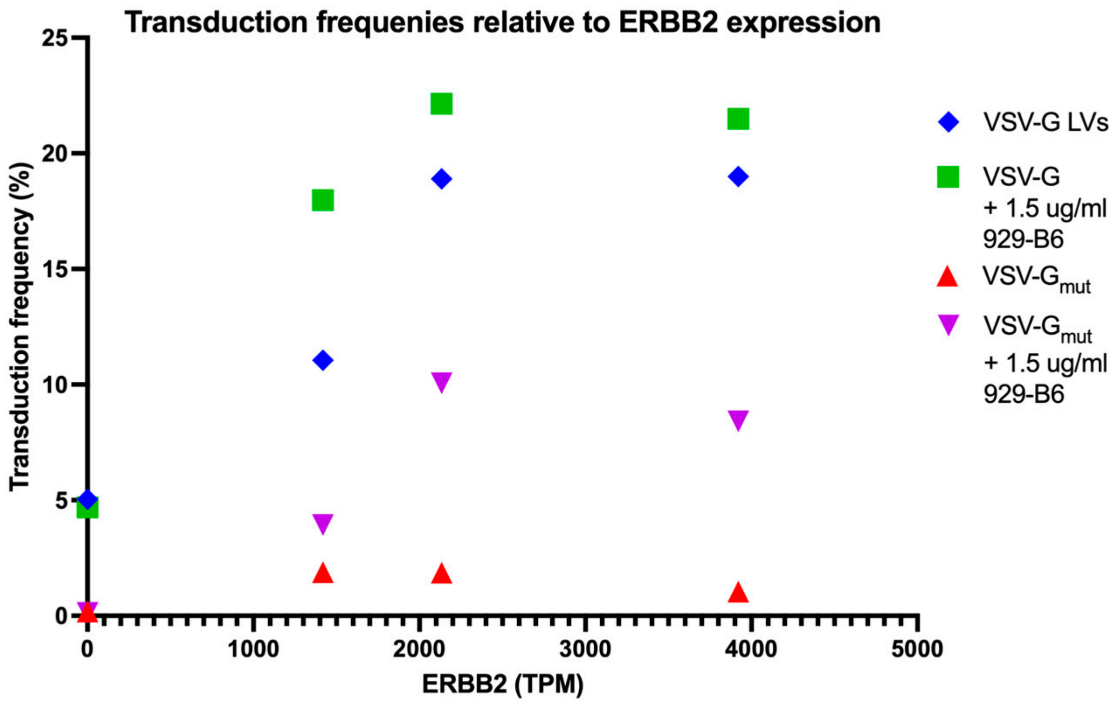


Figure S5. Transduction frequencies of cancer cell lines relative to ERBB2 expression levels (as transcripts per million, according to CCLE 25q3 data).

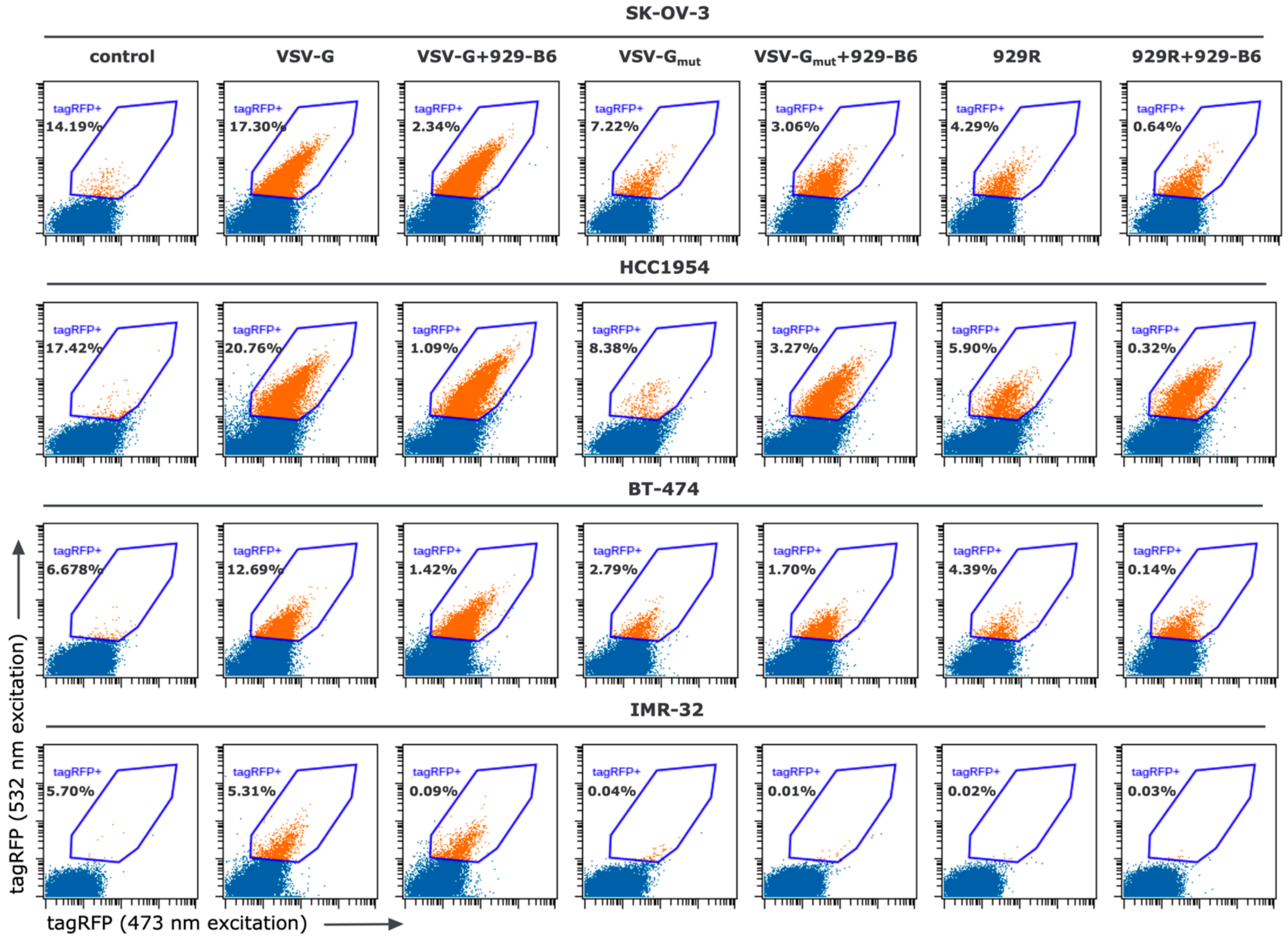


Figure S6. Flow plots of cell line panel transduced with VSV-G, VSV-$G_{mut}$ and VSV-$G_{mut}$+929R-pseudotyped LVs with and without 929-B6 pretreatment, as in Fig. 4C.
